# Socioeconomic inequalities in dental health among middle-aged adults and the role of behavioral and psychosocial factors: evidence from the Spanish National Health Survey

**DOI:** 10.1186/s12939-017-0529-7

**Published:** 2017-02-16

**Authors:** Diego Alberto Capurro, Michael Davidsen

**Affiliations:** 1 Faculty of Dentistry, National University of Asuncion, Yegros 1440 casi 2a. pyda., 1330 Asuncion, Paraguay; 2 National Institute of Public Health, University of Southern Denmark, Øster Farimagsgade 5A, 2nd floor, 1353 Copenhagen K, Denmark

**Keywords:** Socioeconomic inequalities, Dental health, Socioeconomic status, Oral health, Disparities, Psychosocial factors, Behavior, Spain

## Abstract

**Background:**

The goal of this analysis was to describe socioeconomic inequalities in dental health among Spanish middle-aged adults, and the role of behavioral and psychosocial factors in explaining these inequalities.

**Methods:**

This cross-sectional study used survey data from the 2006 Spanish National Health Survey and focused on adults ages 30 – 64. The outcome was dental health status based on the presence of self-reported dental problems. We used education, income, and occupational class as indicators of socioeconomic position and applied logistic regression analysis to estimate associations. We included behavioral and psychosocial variables in the models and compared non-adjusted to adjusted estimates to assess their potential role in explaining socioeconomic gradients.

**Results:**

Results showed clear socioeconomic gradients in dental health among middle-aged adults. The percentage of people who reported more dental problems increased among those with lower levels of education, income, and occupation. These gradients were statistically significant (*p* < .001). Logistic regression showed that groups with lower education, income, and occupation had higher odds of reporting the outcome (*p* < .001). Associations were stronger when considering education as the indicator of socioeconomic position. Substantial unexplained associations remained significant after adjusting the model by behavioral and psychosocial variables.

**Conclusions:**

This study shows significant socioeconomic gradients in dental health among middle-aged adults in Spain. Behavioral and psychosocial variables were insufficient to explain the inequalities described, suggesting the intervention of other factors. Further research should incorporate additional explanations to better understand and comprehensively address socioeconomic inequalities in dental health.

## Background

Dental problems are relevant public health issues due to their prevalence as well as the negative impact they may have on quality of life [1, 2]. In recent decades in Spain and other developed countries, the burden of these dental problems has steadily decreased for the whole population [3, 4]. However, a robust and consistent body of evidence demonstrates that dental problems are unequally distributed across socioeconomic groups: individuals at the lower end of the socioeconomic ladder have a higher burden of dental diseases, compared to those who are better off [5–7]. These inequalities often follow a stepwise gradient pattern, with oral health improving incrementally as socioeconomic position (SEP) improves [8, 9].

In relatively recent years, socioeconomic inequalities in dental health have become a priority in research agendas [5, 10, 11]. Many studies have been conducted to measure inequalities and several mechanisms have been proposed to explain socioeconomic inequalities in dental health. In general, theoretical explanations and models identify a range of intermediate factors through which socioeconomic position affects health: these are material, behavioral and psychosocial factors which operate at different levels and over the life course [12–14]. Evidence suggests health-related behaviors and health care utilization play relevant but limited roles in explaining socioeconomic inequalities in dental health [15–17]. Less is known about the additional contribution of psychosocial factors [18, 19]. In Spain, studies measuring and explaining socioeconomic dental health inequalities at the national level are lacking. This gap of knowledge is particularly relevant for middle-aged adults since this is usually the period of life in which the transition from relatively simple to more complex oral health care needs takes place [20, 21]. Thus, the aims of the present study were to describe socioeconomic inequalities in dental health among middle-aged adults and to assess the role of behavioral and psychosocial factors in explaining the relationship between socioeconomic status and dental health.

## Methods

### Data source

The source of data was the 2006 Spanish National Health Survey—ENSE 2006. ENSE is a program of periodic cross-sectional surveys designed to assess health status in Spain, and it is conducted by the Ministry of Health, Social Services and Equality, in collaboration with the Spanish National Statistics Institute. ENSE 2006 sample was selected to represent the non-institutionalized resident population, and used a complex stratified, multistage probability cluster sample design. ENSE collected data via in-home interviews which included specific questions on demographics, socioeconomic characteristics, health status, health-related behaviours and utilizations of health services. Briefly, the sampling process consisted of three stages. Units in the first stage, census sections, were grouped into strata according to the size of the municipality to which each unit belongs and then randomly selected with a probability proportional to size. Second stage units, households, were selected with equal probability by systematic sampling with random start. Finally, an adult member of each household was randomly chosen by a procedure that assigns the same probability to all the adult members. Detailed information concerning methodology and design is described elsewhere [22]. General household response rate for the survey was 96.11%. This rate was reached by the application of a replacement procedure to those cases in which originally selected households did not participate (31% of the original sample) [23]. The ENSE adult sample included 29,478 individuals (persons ages 16 or more). For this study we used data from the household and adult interview questionnaires and restricted our analysis to adults ages 30 to 64 years.

### Outcome variable

The outcome for the analysis was a proxy indicator of *dental health status based on self-reported dental problems*. Participants were asked dichotomous questions related to the presence of five conditions: caries, gum bleeding, tooth mobility, tooth loss, and prosthetic status. We categorized participants according to the number of dental problems reported: those who reported less than four and those who reported four or five dental problems.

### Socioeconomic variables

In order to capture different aspects of socioeconomic stratification we used three different individual indicators of SEP: education, income, and occupational class [24].

Information on *education* was collected by asking participants to indicate the highest level of education attained. We grouped participants as follows: a) No formal education; b) Primary; c) Secondary; and e) Higher.

Regarding *income*, ENSE 2006 collected information on household net income. Respondents chose their answer from income intervals. Using this information, we calculated an approximation to the *household equivalent net income*, by adjusting the household net income to the composition of each household. We assigned an income value to each participant by calculating the mid-point of the respective income interval and then adjusted this number to the each household composition [25]. Then, four relatively similar-in-size income groups (quartiles) were defined.

*Occupational class* was defined as the occupation of the head of household, which allows occupational class assignation to students, housewives, the unemployed, pensioners, and retired workers. We used the classification recommended by the Spanish Epidemiological Society [26], which comprises five categories: a) Class I (managers of public administration offices and private companies with ten or more employees/professions associated to postgraduate university degrees); b) Class II (managers of companies with fewer than ten employees/professions associated to graduate university degrees, qualified technicians, and others); c) Class III (administrative employees and professionals, personal service and self-employed workers, and supervisors of manual workers); d) Class IV (skilled and semi-skilled manual workers); e) Class V (unskilled workers).

### Behavioural and psychosocial variables

Behavioural variables included were smoking, frequency of sweet consumption, frequency of sugar-sweetened beverage consumption, frequency of toothbrushing, and dental care utilization. For *smoking*, participants were categorized as never, former, and current. *Frequencies of sweet and sugar-sweetened beverage consumptions* were based on approximate weekly consumptions: a) less than three times per week; b) three or more times per week but not daily; c) daily. For *frequency of toothbrushing* respondents were categorized into three groups: a) at least twice a day; b) once a day; c) less than once a day. *Dental care utilization* was based on dental visits within the last 12 months.

Psychosocial variables included were psychological distress, perceived family functioning, social support, work-related stress, and job satisfaction. To assess psychological distress ENSE includes the Spanish adaptation of the Goldberg’s General Health Questionnaire (GHQ-12) [27, 28]. Values range from 0 – 12 points, with higher values indicating higher level of distress. We categorized participants as with Low (0 – 3), Moderate (4 – 7), and High psychological distress (8 – 12). For perception on family functioning, the five-question Family APGAR questionnaire was used [29, 30]. Response options were designed to describe the frequency of feeling satisfied with each parameter on a 3-point scale ranging from 0 (hardly ever) to 2 (almost always). The scale is scored by summing the values for the five items for a total score that ranges from 0 – 10. We categorized participants as those with Poor (0 – 3), Fair (4 – 6) and Good (7 – 10). Social support was measured using the Duke-UNC Functional Social Support Questionnaire, which is an eleven-item instrument to measure the strength of a person´s social support network [31, 32]. Responses to each question are scored on a 1 – 5 scale. The final score range from 11 – 55 points (the higher the score, the greater the perceived social support). Scores were categorized in Poor (11 – 25), Fair (26 – 40) and Good (41 – 55). Finally, for work-related stress and job satisfaction participants were asked to assess their level of stress and satisfaction using single questions (ranges 1 – 7). Work-related stress scores were grouped in Low (1 – 3), Moderate (4 – 5) and High (6 – 7); and job satisfaction in Low (1 – 3), Moderate (4 – 5) and High (6 – 7).

Other covariates included in the analysis were age, sex, area of residence, and marital status.

### Data analysis

Data description was performed through descriptive statistics. Percentage differences were tested with Person chi-square. Associations between each socioeconomic variable and dental health are expressed as crude and adjusted odds ratios (OR), derived from logistic regression models. Three models were built separately for each indicator of SEP. The first model included demographic variables (age, sex, marital status and area of residence). The second model was adjusted by demographic and behavioural variables, and the third model additionally included psychosocial variables. To assess how much of the associations were explained by behaviour and psychological factors, we compared unadjusted (first model) and adjusted ORs (third model) and estimated the percentage reduction in odds, defined as the ratio of the difference between the unadjusted and adjusted ORs to the difference between the unadjusted OR and one, multiplied by 100) [33]. In a final common model, we included simultaneously all socioeconomic factors to assess for further changes and independent effects.

Associations were considered as significant when *p* < 0.05. In order to evaluate the adequacy of presenting separated models, tests for no interaction (Wald test) were performed between each socioeconomic variable and sex and age. All estimates are presented with their respective 95% confidence intervals. Missing data is detailed in the result section. For those variables with missing data >10% (income, work related stress, and job satisfaction) we applied multiple imputation (MI) [34]. MI is a Monte Carlo technique in which the missing values are replaced by *m* > 1 simulated versions, where *m* is typically small (we computed five simulations). We included all the variables considered in this study as predictors of the imputed values. Each of the simulated datasets was analyzed by standard methods, and results combined to produce estimates and confidence intervals that incorporate missing-data uncertainty [34, 35]. Relative frequencies are reported as weighted percentages (sampling weights applied). All analyses were performed using weighted data and standard errors adjusted to the complex survey design. Data analysis was conducted using SPSS v.18.0 and Stata v.14.0.

## Results

The study sample consisted of 17,602 individuals. Table 1 presents the population distribution with regards to the variables considered. Non-response rates were in general low, but higher for income (14.2%), work-related stress (35.8%) and job satisfaction (35.7%). Those who reported four or five dental problems accounted for 9.9% of the population.

**Table 1:** Study sample characteristics— adults ages 30 – 64 years

		n	% (CI 95%)
Sex			
	Male	7,004	50.1 (49.0–51.2)
	Female	10,598	49.9 (48.8–51.0)
Marital Status			
	Single	3,373	19.8 (18.9–20.8)
	Married	12,139	71.4 (70.4–72.4)
	Separated/Divorced	1,365	6.0 (5.6–6.5)
	Widowed/Widower	676	2.3 (2.0–2.5)
	Missing	49	0.5 (0.3–0.7)
Area of Residence			
	Urban	13,116	79.0 (77.6–80.2)
	Semi-urban	3,192	15.5 (14.1–17.0)
	Rural	1, 294	5.6 (4.7–6.6)
Level of education			
	No formal education	1,097	5.9 (5.4–6.5)
	Primary	5,851	31.6 (30.5–32.9)
	Secondary	7,209	41.4 (40.2–42.6)
	University	3,356	20.1 (18.9–21.2)
	Missing	89	1.0 (0.7–1.3)
Equivalent net income			
	Quartile 1	3,473	21.6 (20.5–22.8)
	Quartile 2	3,656	20.1 (19.2–21.0)
	Quartile 3	5,000	25.8 (24.8–26.9)
	Quartile 4 (highest)	3,548	18.3 (17.3–19.3)
	Missing	1,925	14.2 (12.9–15.5)
Occupational class			
	I (highest)	1,886	11.3 (10.5–12.2)
	II	1,921	10.8 (10.1–11.5)
	III	4,349	23.6 (22.7–24.5)
	IV	7,064	39.9 (38.7–41.0)
	V	2,122	12.5 (11.8–13.4)
	Missing	260	1.9 (1.6–2.3)
Self-reported dental problems^a^			
	<four dental problems	15,700	87.4 (86.6–88.3)
	≥four dental problems	1,655	9.9 (9.3–10.6)
	Missing	247	2.6 (2.1–3.3)
Dental visits in the last 12 months			
	Yes	7,424	40.4 (39.4–41.5)
	No	10,178	59.6 (58.5–60.6)
Smoking			
	Never	7,782	42.7 (41.6–43.7)
	Former	4,056	23.3 (22.4–24.2)
	Current	5,764	34.1 (33.1–35.1)
Frequency of sweet consumption per week			
	Less than three times	9,282	52.7 (51.5–53.9)
	Three times or more	2,102	12.0 (11.3–12.8)
	Daily	5,904	31.8 (30.6–33.0)
	Missing	314	3.5 (2.8–4.2)
Frequency of sweet beverage consumption per week			
	Less than three times	13,790	73.5 (72.4–74.7)
	Three times or more	1,200	7.7 (7.1–8.3)
	Daily	2,290	15.3 (14.4–16.2)
	Missing	322	3.5 (2.9–4.3)
Frequency of toothbrushing			
	At least twice a day	12, 216	65.8 (64.6–67.0)
	Once a day	3,713	22.6 (21.6–23.5)
	Less than once a day	1,376	8.9 (8.3–9.6)
	Missing	297	2.7 (2.2–3.3)
Psychological distress			
	Good	14,306	79.3 (78.1–80.5)
	Fair	1,817	9.6 (9.0–10.2)
	Poor	798	4.3 (3.9–4.7)
	Missing	681	6.9 (5.9–8.1)
Social support			
	Good	14,516	79.7 (78.5–80.9)
	Fair	2,087	11.8 (11.0–12.6)
	Poor	209	1.0 (0.8–1.2)
	Missing	790	7.5 (6.6–8.6)
Family function			
	Good	15,858	88.8 (87.8–89.7)
	Fair	901	4.6 (4.2–5.1)
	Poor	265	1.1 (0.9–1.4)
	Missing	578	5.4 (4.6–6.4)
Work-related stress			
	Low	3,372	19.3 (18.5–20.2)
	Moderate	5,090	29.9 (28.9–30.9)
	High	2,527	15.0 (14.2–15.8)
	Missing	6,613	35.8 (34.7–36.9)
Job satisfaction			
	High	4,703	26.3 (25.4–27.3)
	Moderate	4,755	28.5 (27.5–29.5)
	Low	1,537	9.4 (8.8–10.1)
	Missing	6,607	35.7 (34.6–36.8)

^a^Based on self-reported dental problems: ‘Presence of tooth decay’ (30.7%, CI =29.7–31.8), ‘Missing tooth’ (76.7%, CI = 75.6–77.8), ‘Gum bleeding’ (22.6%, CI = 21.6–23.5), ‘Tooth mobility’ (7.8%, CI = 7.2–8.3), and ‘Missing tooth with no prosthetic replacement’ (56%, CI = 54.8–57.2)

The distribution of people who reported four or five dental problems with respect to socioeconomic, behavioral and psychosocial variables is detailed in Table 2. Clear and consistent socioeconomic gradients were observed. For the three indicators of SEP gradients were significant (*p* < .001). The prevalence of the outcome among the people without formal education was more than five times higher than the group with university background (22 vs. 4%). Similar differences existed when considering income or occupational class as SEP indicators (16.4 vs. 5.2% for income and 14.5 vs. 4.4% for occupational class). As expected, the prevalence of the outcome was lower among non-smoker and those who reported frequent toothbrushing and dental visits in the last 12 months. Psychosocial gradients were also observed: outcome prevalences increase with higher levels of psychological distress, as well as with lower levels of social support, family function and job satisfaction (*p* < .001).

**Table 2:** Distribution of people reporting four or five dental problems by socioeconomic, behavioral and psychosocial variables— adults ages 30 – 64 years

		≥4 dental problems	
		*n*	% (CI 95%)
Level of education^+^			
	No formal education	232	21.9 (18.8–25.5)
	Primary	730	13.6 (12.4–14.9)
	Secondary	560	8.9 (8.0–10.0)
	University	127	4.0 (3.2–5.0)
Equivalent net income^+a^			
	Quartile 1	576	16.4 (14.7–18.2)
	Quartile 2	419	10.8 (9.4–12.3)
	Quartile 3	423	8.4 (7.4–9.5)
	Quartile 4 (highest)	237	5.2 (4.3–6.1)
Occupational class^+^			
	I (highest)	83	4.4 (3.3–5.9)
	II	117	6.7 (5.3–8.5)
	III	356	9.0 (7.9–10.4)
	IV	784	12.2 (11.1–13.4)
	V	290	14.5 (12.5–16.8)
Frequency of sweet consumption per week^+^			
	Less than three times	904	10.9 (10.0–11.9)
	Three times or more	161	7.5 (6.2–9.2)
	Daily	579	10.4 (9.3–11.5)
Frequency of sweet beverage consumption per week^+^			
	Less than three times	1,208	9.4 (8.7–10.2)
	Three times or more	140	12.1 (9.8–14.9)
	Daily	296	13.7 (11.7–15.9)
Smoking^+^			
	Never	692	8.9 (8.0–9.8)
	Former	414	11.7 (10.4–13.3)
	Current	549	10.8 (9.7–12.1)
Frequency of toothbrushing^+^			
	At least twice a day	866	7.6 (6.9–8.3)
	Once a day	461	12.5 (11.1–13.9)
	Less than once a day	309	24.5 (21.4–27.8)
Dental visits in the last 12 months^+^			
	Yes	562	8.6 (7.7–9.6)
	No	1,093	11.3 (10.5–12.2)
Psychological distress^+^			
	Good	1,186	8.9 (8.3–9.7)
	Fair	261	16.5 (14.1–19.3)
	Poor	160	21.0 (17.5–25.0)
Social support^+^			
	Good	1,297	9.7 (9.0–10.5)
	Fair	272	14.0 (12.1–16.2)
	Poor	43	22.3 (15.4–31.2)
Family function^+^			
	Good	1,444	9.9 (9.2–10.6)
	Fair	131	15.0 (12.0–18.6)
	Poor	47	24.0 (16.7–33.4)
Work-related stress^a^			
	Low	541	9.88 (8.53–11.23)
	Moderate	716	9.82 (8.80–10.84)
	High	398	11.55 (9.95–13.15)
Job satisfaction^+a^			
	High	547	8.41 (7.26–9.57)
	Moderate	753	10.81 (9.75–11.87)
	Low	355	12.79 (10.88–14.70)

^a^Estimates calculated with multiply imputed data

^+^ *p* < .005

Finally, Table 3 shows the associations between SEP indicators and the outcome, derived from ten different logistic regression models. Associations adjusted by demographic factors are presented under ‘Model 1’ (three models, one model for each SEP indicator). The odds of reporting more dental problems decreased with levels of education and income, and increased among lower occupational classes. The strongest associations were found between education and the outcome: the OR for education was 6.54 (CI_95%_ 4.80 – 8.91), higher than the ones observed for income and occupation (3.62 (CI_95%_ 3.17 – 4.07) and 3.61 (CI_95%_ 2.53 – 5.16), respectively). Estimates after additional adjustment by behavioral variables only, and by behavioral and psychosocial variables are shown under ‘Model 2’ and ‘Model 3’, respectively. The magnitude of the associations decreased but remained significant after these adjustments: 4.38 (CI_95%_ 3.68 – 5.08) for education, 2.46 (CI_95%_ 2.06 – 2.87) for income and 2.35 (CI_95%_ 1.77 – 2.92) for occupation. Behavioral and psychosocial variables accounted for 39% of the educational gap between those with no formal education and those with university education (the unadjusted OR = 6.54 (Model 1) decreased to OR = 4.38 after adjustments (Model 3)), 44% of the income gap between Quartiles 1 and 4 (from the unadjusted OR = 3.62 (Model 1) to OR = 2.46 after adjustments (Model 3)), and 48% of the occupation gap between Class I and V (OR = 3.61 in model in Model 1 to OR = 2.35 in Model 3). After the inclusion of the three socioeconomic variables simultaneously in the final model (Model 4) education and income gaps remained significant (OR = 2.94 and OR = 1.80; respectively, *p* < .001) and significant associations between occupational class and the outcome disappeared.

**Table 3:** Associations between SEP and reporting four or five dental problems—adults ages 30 – 64 years

	Model 1^a^		Model 2^b^		Model 3^c^		Model 4^d^	
	OR (CI_95%_)	*p*	OR (CI_95%_)	*p*	OR (CI_95%_)	*p*	OR (CI_95%_)	*p*
Level of Education								
University^e^	----	---	----	---	----	---	----	---
Secondary	2.33 (1.80–3.01)	.000	2.05 (1.58–2.66)	.000	1.96 (1.59–2.33)	.000	1.62 (1.26–1.97)	.000
Primary	3.69 (2.86–4.77)	.000	2.82 (2.16–3.67)	.000	2.57 (2.14–3.00)	.000	1.89 (1.48–2.29)	.000
No formal education	6.54 (4.80–8.91)	.000	4.58 (3.31–6.34)	.000	4.38 (3.68–5.08)	.000	2.94 (2.31–3.57)	.000
	*n* = 17,267 (98%)		*n* = 16,920 (96%)		*n* = 16,198 (92%)			
	F–adjusted = 0.66 (*p* = 0.746)		F–adjusted = 0.95 (*p* = 0.481)		F-adjusted = 1.06 (*p* = 0.388)			
	log pseudolikelihood = −6741427.9		log pseudolikelihood = −6364312.7		log pseudolikelihood = −5910612.5			
	Model 1^a^		Model 2^b^		Model 3^c^			
	OR (CI_95%_)	*p*	OR (CI_95%_)	*p*	OR (CI_95%_)	*p*		
Household Equivalent								
Income	----	---	----	---	----	---	----	---
Quartile 4 (highest)^e^	1.68 (1.37–1.98)	.000	1.54 (1.24–1.84)	.000	1.49 (1.19–1.79)	.002	1.25 (0.96–1.53)	.089
Quartile 3	2.20 (1.82–2.58)	.000	1.80 (1.45–2.15)	.000	1.73 (1.40–2.07)	.000	1.35 (1.05–1.65)	.022
Quartile 2	3.62 (3.17–4.07)	.000	2.77 (2.36–3.18)	.000	2.46 (2.06–2.87)	.000	1.80 (1.43–2.16)	.000
Quartile 1								
	*n* = 17,332 (98%)		*n* = 16,979 (96%)		*n* = 16,246 (92%)			
	log pseudolikelihood = −6807884.4		log pseudolikelihood = −6413004.9		log pseudolikelihood = −5969405.4			
	F-adjusted = 0.56 (*p* = 0.827)		F-adjusted = 0.99 (*p* = 0.445)		F-adjusted = 0.95 (*p* = 0.480)			
Occupational class								
I (highest)^e^	----	---	----	---	----	---	----	---
II	1.56 (1.06–2.31)	.025	1.37 (0.92–2.04)	.121	1.35 (0.88–1.82)	.142	1.09 (0.67–1.51)	.674
III	2.08 (1.48–2.93)	.000	1.78 (1.26–2.52)	.002	1.60 (1.15–2.04)	.009	1.10 (0.73–1.48)	.587
IV	2.94 (2.13–4.07)	.000	2.27 (1.63–3.17)	.000	2.13 (1.63–2.62)	.000	1.26 (0.86–1.65)	.208
V	3.61 (2.53–5.16)	.000	2.55 (1.77–3.66)	.000	2.35 (1.77–2.92)	.000	1.25 (0.81–1.69)	.270
	*n* = 17,111 (97%)		*n* = 16,778 (95%)		*n* = 16,068 (91%)		*n* = 16,023 (91%)	
	log pseudolikelihood = −6790156.0		log pseudolikelihood = −6382456.4		log pseudolikelihood = −5941464.8		log pseudolikelihood = −5823027.6	
	F-adjusted = 0.28 (*p* = 0.979)		F-adjusted = 0.60 (*p* = 0.798)		F-adjusted = 0.90 (*p* = 0.527)		F-adjusted = 1.52 (*p* = 0.137)	

Models 1, 2 and 3 were built separately for each indicator of SEP. Model 4 is one single model with the three indicators of SEP included simultaneously

^a^Model 1: adjusted by demographic variables (age in groups, sex, area of residence, and marital status)

^b^Model 2: adjusted by demographic and behavioral variables (smoking, sweet and sweetened beverage intake, toothbrushing, and dental visits)

^c^Model 3: adjusted by demographic, behavioral, and psychosocial variables (mental health, social support, family function, work-related stress, and job satisfaction. Work-related stress and job satisfaction with imputed data)

^d^Model 4: adjusted by demographic, behavioral, psychosocial, and socioeconomic variables (income, work-related stress, and job satisfaction with imputed data). To avoid multicollinearity, the degree of correlation between socioeconomic variables was measured. The highest correlation found (between education and occupational class) was moderate (Spearman’s rho = 0.47; *p* < 0.001)

^e^Reference category

## Discussion

Results show important socioeconomic differences in the distribution of dental health status among middle-ages adults in Spain. The prevalence of people reporting four or five dental problems steadily decreased as levels of education and income increased and occupation decreased. Education showed the strongest associations with the outcome. When assessing the role of potential mediators such as behavioral and psychosocial characteristics, associations did not disappear. When including the three indicators of SEP in the model, attenuated education and income gradients remained and the occupation-related gradient disappeared.

Socioeconomic inequalities in dental health reported here are congruent with findings from other high-income countries [5]. The finding that health-related behaviors have a limited role in explaining the observed social inequalities in dental health is consistent with previous research [16, 17, 36]. Furthermore, inequalities remained even after adjustment by psychosocial variables. Associations between selected psychosocial variables and oral health have been measured [37–40] but their role in explaining socioeconomic oral health inequalities remains scarcely explored [18, 19]. Our study found psychosocial differences in the outcome but the role of such differences in explaining socioeconomic gradients was limited. Further decreases in the association measures were observed when SEP indicators were included in the models and, moreover, the occupation gradient disappeared when adjusted by income and education. This is expectable since education, income, and occupation are correlated and mediate each other’s effect over the life course, thus suggesting the critical role of more common structural factors for inequality explanation.

These findings emphasize the need to explore additional pathways to fully explain the gradients. Current and past material circumstances, childhood disease experiences, early-life unequal exposure to risk factors through social and environmental circumstances, and life course experiences have been proposed as other potential mechanisms [13, 14]. In addition, structural supply factors also deserve special attention in the generation of inequalities in Spain: despite progressive implementation of publicly-financed child dental care programs across the country increased access to dental care for this population [41], dental care for adults is almost exclusively provided by the private sector and mainly financed through out-of-pocket payments. In 2011, out-of-pocket dental expenditure represented 97.4% of the total dental expenditure in the country [42], and dental care was the highest private health expenditure among Spanish households, representing more than 40% of households’ health expenditure [43].

Along our findings limitations should be noted. Ours was a cross-sectional study and therefore causal relationships cannot be concluded. Income was reported in intervals and irrespective of household composition. To partially account for this we adjusted income by household members. Moreover, our study focused only on individual SEP. Some studies demonstrated the importance of including area-based indicators when describing socioeconomic inequalities in dental health [44, 45]. Thus, it is possible that relevant dimensions of socioeconomic inequalities were not reflected in our study. Further studies incorporating census data are needed to explore further inequalities.

There also may be other potential psychosocial factors not included in our study. Social capital and sense of coherence may play additional roles in the psychosocial explanation of inequalities [37, 40, 46] and therefore should be subject of future research.

Estimations of adjusted associations were calculated using imputed data for income, work related stress, and job satisfaction. While missing cases regarding income represented a relatively low proportion of the study sample, information on work-related stress and job satisfaction was collected only for current workers and missing cases represented a larger proportion. To account for this, we applied MI. To ensure MI did not lead to bias results we performed sensitivity analyses with complete cases. Magnitude and direction of inequalities were very similar to the ones presented here (results upon request).

Also, it is important to note the oral health outcome was based on self-reported dental conditions. Self-reported problems are subject to information bias and do not account for the severity of each problem reported. However, there is evidence that supports self-reported dental conditions as valid and reliable indicators of dental health [47–49] and with good correlates with clinical findings, especially for those problems that are easy to observe by respondents [47]. Some conditions such as caries, dental mobility and gum bleeding are not likely to be detected in early stages but, when the condition is severe, self reports could be considered a reasonable and useful screening to detect poor status and need of care [47, 50]. The cutpoint of dental problems reported was four. With this, we tried to capture those participants with more dental problems; however, more problems reported does not necessarily mean poorer dental health. Different cutpoints may lead to different results and sensitivity analysis was conducted to assess for these changes (results upon request). Different cutpoint selection affected the magnitude and precision of the associations, but not the patterns of the associations found. People with five dental problems accounted for 1.7% of the population, and those with three or more dental problems accounted for 33%. Thus, when the coutpoint was five dental problems the magnitude of the odds ratios was greater and less precise (wider confidence intervals). In contrast, when the cutpoint was three or more dental problems associations decreased and confidence intervals narrowed. In both cases clear gradients were observed and associations remained after adjustments. Unfortunately, ENSE does not include other indicators of dental health meaning comparisons with other outcomes are not feasible. In this sense, it is strongly recommended the inclusion of other indicators such as self-assessed number of teeth and self-perceived oral health as more standardized indicators of oral health status in adult population.

Finally, the use of relatively old data may pose some limitation to our work. In comparison to the more recent survey wave (2011), ENSE 2006 collected information on more psychosocial and demographic variables (i.e. family function and area of residence), which are factors known to affect oral health [14]. Since the time the survey was conducted there has been no evidence on important changes in the provision of dental care or in other major policies which are expected to change behaviors in the study population. Therefore we consider our results relevant for the current Spanish context.

## Conclusions

This study shows socioeconomic inequalities in dental health among middle-age adults in Spain. Consistent with previous findings, behaviors have a partial explanatory role. We also found that psychosocial explanations were also insufficient. These findings highlight the need to assess other potential explanations of the inequalities described.

## Abbreviations

ENSE: Spanish National Health Survey
SEP: Socioeconomic position
OR: Odds ratios
MI: Multiple imputation
